# Melphalan as a Promising Treatment for BRCA-Related Ovarian Carcinoma

**DOI:** 10.3389/fonc.2021.716467

**Published:** 2021-07-21

**Authors:** Vincenza Conteduca, Emanuela Scarpi, Alberto Farolfi, Nicole Brighi, Lorena Rossi, Giorgia Gurioli, Cristian Lolli, Giuseppe Schepisi, Sara Bleve, Caterina Gianni, Alessandra Virga, Amelia Altavilla, Salvatore Luca Burgio, Cecilia Menna, Ugo De Giorgi

**Affiliations:** ^1^ Department of Medical Oncology, IRCCS Istituto Romagnolo per lo Studio dei Tumori (IRST) “Dino Amadori”, Meldola, Italy; ^2^ Unit of Biostatistics and Clinical Trials, IRCCS Istituto Romagnolo per lo Studio dei Tumori (IRST) “Dino Amadori”, Meldola, Italy; ^3^ Biosciences Laboratory, IRCCS Istituto Romagnolo per lo Studio dei Tumori (IRST) “Dino Amadori”, Meldola, Italy

**Keywords:** melphalan, ovarian cancer, BRCA, platinum resistance, survival

## Abstract

**Introduction:**

Melphalan, as a bifunctional alkylating agent has been shown to be selectively efficient in BRCA-deficient case reports of epithelial ovarian cancer (EOC). The clinical benefit of melphalan on unselected platinum-resistant EOC population and stratified by BRCA status has not been clearly elucidated. We aimed to determine the response to melphalan in patients with recurrent EOC after platinum-based therapy.

**Material and Methods:**

This retrospective observational study included patients with recurrent EOC treated with melphalan between February 2007 to July 2020. Eligibility criteria included having a histological confirmation of EOC, previous treatment with carboplatin plus paclitaxel regimens, and disease recurrence during treatment with or within 6 months of the end of the platinum-based chemotherapy.

**Results:**

A total of 75 platinum-resistant EOC patients were enrolled. Median age was 69 years (range 41-82). Median of previous therapies before melphalan was 4 (range 1-7). We observed a median follow-up of 32 months (range 1-62), progression-free survival (PFS) and overall survival (OS) of 3.6 months (range 2.9-4.7) and 9.5 months (range 8.0-14.1), respectively. In the whole population, 1 complete response, 6 partial responses and 37 stable diseases were registered with an overall clinical benefit rate of 58.7%. In BRCA1/2 mutant patients, we showed a significant longer PFS compared to BRCA1/2 wild type patients (6.2 *versus* 2.6 months; hazard ratio (HR) 0.25, 95% confidence interval (CI) 0.10-0.61; *p*=0.002). Moreover, a trend was seen for BRCA1/2 mutants to have a better OS (25.9 *versus* 8.0 months; HR 0.38; 95% CI 0.12-1.19; *p*=0.097).

**Conclusions:**

Our study represents the largest cohort of heavily-pretreated EOC patients receiving melphalan treatment. Here, we report a considerable clinical activity of melphalan chemotherapy, more evident in a subset of BRCA1/2 mutated patients. Prospective studies to validate these findings are warranted.

## Introduction

Ovarian cancer is a leading cause of death from gynaecologic cancers worldwide (1). Despite optimal debulking surgery, appropriate first-line chemotherapy based on taxane-platinum doublets and combination/maintenance therapy with bevacizumab or poly(adenosine diphosphate-ribose) polymerase inhibitors (PARPi), approximately 60-70% of patients eventually relapse (2, 3). For recurrent patients, a rate of response more than 60% is reported in platinum-sensitive patients (occurring at least 6 months after last treatment completion) receiving platinum-based combination chemotherapy, whilst the response rate dramatically drops to less than 20% (4) in platinum-resistant women (recurring within 6 months after the last therapy) who can receive several drugs characterized by different mechanisms of action and, in general, a modest activity, such as topotecan (5), gemcitabine (6), liposomal doxorubicin (7), oral etoposide (8), ifosfamide (9) and oxaliplatin plus leucovorin and 5-fluorouracil (FOLFOX-4) (10). Thus, there is an urgent need to identify agents active in this group of EOC platinum-resistant patients.

**Figure 1:**
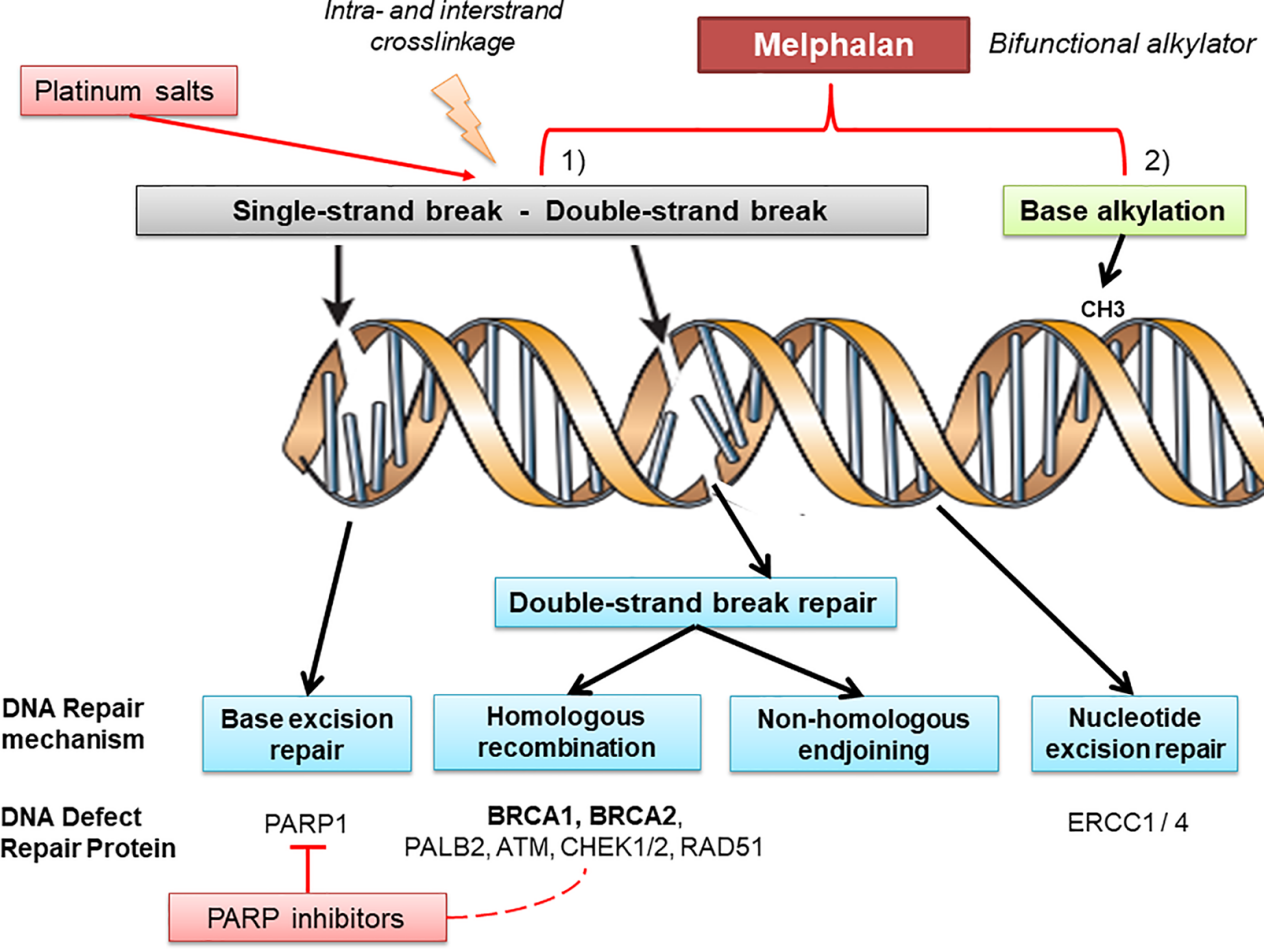
Melphalan and DNA repair mechanisms. DNA is continually exposed to a series of insults that cause a range of lesions, from single-strand breaks to base alkylation events. Several mechanisms of DNA repair (such as base excision repair, homologous recombination, non-homologous endjoining, and nucleotide excision repair) can be involved recruiting different key proteins which belong to pathways used in the therapeutic strategy in ovarian cancer. Alkylating agents, platinum salts, and PARP inhibitors are particularly effective in DNA defect repair deficient tumors, albeit through different molecular mechanisms. Melphalan is a bifunctional alkylating agent that produces intra- and inter-strand cross-links in double-strand DNA and provides base alkylation, whereas platinum mainly generates intra-strand crosslinks through platinum coordinated complexes and PARP1 inhibitors block base excision repair leading to single-strand breaks. As a different spectrum of DNA damage is produced by each drug, it is possible that the DNA damage produced by melphalan may be more reliant on BRCA protein products for repair.

Melphalan is a nitrogen mustard-like alkylating agent, administered orally or parenterally and mainly used for the treatment of multiple myeloma. Very little information is available regarding the use of melphalan for the treatment of epithelial ovarian cancer (EOC), thus providing controversial results to date (11, 12). Additionally, melphalan, considered as a bifunctional alkylating agent that induces inter- and intra-strand DNA cross-links, has been shown to be selectively efficient in BRCA-defıcient case reports of EOC (13, 14) (**Figure 1**).

Here we evaluated the efficacy and toxicity of melphalan in heavily-pre-treated platinum-resistant EOC. In addition, through an exploratory analysis, we aimed to show an increased sensitivity to melphalan in patients harbouring BRCA1/2-defıciency.

## Materials and Methods

### Study Population

This is a retrospective single-centre case series of patients with ovarian cancer receiving melphalan from February 2007 to July 2020. Eligibility criteria were: histological confirmation of epithelial ovarian cancer, previous treatment with carboplatin plus paclitaxel regimen, and disease recurrence during treatment within 6 months from the end of the platinum-based chemotherapy. Additional eligibility criteria included: Eastern Cooperative Oncology Group (ECOG) performance status 0–2, and adequate cardiac, renal, hepatic and bone marrow function. Metastatic disease was documented by bone scan, computed tomography or magnetic resonance imaging. For each patient, we extracted clinical-pathologic features, treatment history and outcomes with follow-up data from medical records. Moreover, we collected molecular data including somatic and/or germ line BRCA1/2 status from each subject, when available. The study was approved by the Institutional Review Board of IRCCS Istituto Romagnolo per lo Studio dei Tumori (IRST) “Dino Amadori”, Meldola, Italy.

### Treatment and Evaluation

Melphalan (2 mg/tablet, Alkeran™; Excella GmbH; Feucht, Germany) was orally administered at dosage of 0,20 mg/Kg daily for 5 consecutive days monthly until evidence of either progressive disease (PD) or unacceptable toxicity.

Patients were evaluated monthly for safety and dosing compliance. Renal, liver and bone marrow function were assessed at every cycle, while cancer antigen 125 (CA-125) and radiographic evaluation were left to the discretion of the treating physician, but were usually performed after at least every three months during treatment.

Tumour response was usually evaluated every three cycles by repeating baseline assessments using imaging studies (computed tomography and/or magnetic resonance imaging) according to the Response Evaluation Criteria in Solid Tumours (RECIST) for patients with measurable disease (15). CA-125 was evaluated in recurrent disease using CA-125 response criteria developed by the Gynaecologic Cancer InterGroup (16). Toxicity was graded using the National Cancer Institute Common Terminology Criteria for Adverse Events (CTCAE), version 4 (17).

### Statistical Analysis

All data were analyzed by descriptive statistics. Relationships between patients’ characteristics were tested using the Chi-square test for categorical variables and the median test for continuous variables. The Kaplan-Meier method was used to estimate progression-free survival (PFS) and overall survival (OS), with two-sided 95% confidence intervals (95% CI). PFS was defined as the time from the start of melphalan until disease progression or last tumour evaluation or death from any cause. OS was defined as the time from the start of melphalan until death from any cause or last follow-up. Survival curves were compared using the log-rank test. All statistical analyses were carried out with SAS statistical software, version 9.4 (SAS Institute, Cary, NC, USA). A two-sided *p*-value < 0.05 was deemed statistically significant for all the analyses.

## Results

### Patients’ Characteristics

Between February 2007 and July 2020, a total of 75 patients were eligible. Median age was 69 years (range 41-82). Median of previous therapies before melphalan was 4 (range 1-7). At the time of study entry, all patients were defined as resistant to the last platinum treatment. We excluded EOC patients receiving concurrent use of other anticancer agents or treatments. The majority of the patients (~90%) had high-grade serous type. Forty-three patients (57.3%) had initial International Federation of Gynecology and Obstetrics (FIGO) stage III, and 66 (88.0%) underwent primary or interval debulking surgery. Patient characteristics are summarized in **Table 1**.

**Table 1:** Patient characteristics.

	N (%)
**Median age at start of melphalan**, years (range)	69 (33-87)
**Histology**	
Serous	67 (89.3)
No-serous	8 (10.7%)
**FIGO stage at presentation**	
II	9 (12.0)
III	43 (57.3)
IV	23 (30.7)
**Grade**	
G1/2	6 (9.8)
G3	55 (90.2)
Unknown	14
**Primary debulking surgery**	
No	9 (12.0)
Yes	66 (88.0)
**BRCA status**	
Wild-type	25 (69.4)
Mutated	11 (30.6)
Not available/Unknown	39
**ECOG PS**	
0-1	67 (89.3)
≥2	8 (10.7)
**Median baseline Ca125**, ng/mL (range)	287.3 (11.1-10535)
**Pre-treatment hemoglobin**, g/dl	
>12.5^#^	18 (25.7)
≤12.5	52 (74.3)
Unknown	5
**Pre-treatment NLR**	
<3	34 (50.7)
≥3	33 (49.3)
Unknown	8
**Pre-treatment PLR**	
<210	34 (50.0)
≥210	34 (50.0)
Unknown	7
**Number of previous therapies before Melphalan**	
2	15 (20.0)
3	17 (22.7)
4	15 (20.0)
5	12 (16.0)
6	9 (12.0)
≥7	7 (9.3)
**Median number of cycles of Melphalan** (range)	3 (1-22)
**Starting dose of Melphalan**	
95-100%	32 (42.7)
75-94%	32 (42.7)
<75%	11 (14.6)
**Dose reduction of Melphalan during treatment**	
No	54 (72.0)
Yes	21 (28.0)
**Median follow-up**, months (range)	32 (1-62)
**Median PFS**, months (95% CI)	3.6 (2.9-4.7)
**Median OS**, months (95% CI)	9.5 (8.0-14.1)
**Tumor response**, N (%)	
CR	1 (1.3)
PR	6 (8.0)
SD	37 (49.4)
PD	31 (41.3)
Ca125 response^*^, N (%)	20 (26.7)
**Patients receiving new treatment after progression**, N (%)	38 (50.7)
**Median number of therapies after melphalan** (range)	2 (1-5)

^#^Upper normal value.

*According to Rustin’s criteria.

CI, confidence interval; CR complete response; ECOG, Eastern Cooperative Oncology Group; FIGO International Federation of Gynecology and Obstetrics; N, number; NLR, neutrophil-to-lymphocyte ratio; OS, overall survival; PFS, progression-free survival; PLR platelet-to-lymphocyte ratio; PD, progression disease; PR, partial response; PS, performance status; SD, stable disease.

### Clinical Outcomes in Overall Melphalan-Treated Patients and Carriers of BRCA1/2 Mutations

The median follow-up was 32 months (range 1-62). All patients had measurable disease. In the whole population, 1 complete response (CR), 6 partial responses (PR) and 37 stable diseases (SD) were registered with an overall clinical benefit rate (CR+PR+SD) of 58.7%. A CA-125 response was observed in 20 (26.7%) melphalan-treated patients and it was not associated with objective radiological response (CR or PR).

Median PFS and OS were of 3.6 months (range 2.9-4.7) and 9.5 months (range 8.0-14.1), respectively (**Table 1**). BRCA status was associated with outcomes at univariate analysis. On the other hand, univariate analyses did not identify any other significant factors (including age, debulking surgery, FIGO stage, ECOG performance status, pre-treatment CA-125, hemoglobin, neutrophil-to-lymphocyte ratio (NLR) and platelet-to-lymphocyte ratio (PLR) predicting PFS and OS (**Table 2**).

**Table 2:** Univariate analysis of progression-free survival and overall survival.

	PFS						OS					
	N. pts	N. events	Median PFS (mo) (95% CI)	p	HR (95% CI)	p	N. pts	N. events	Median OS (mo) (95% CI)	p	HR (95% CI)	p
**Age**, years												
<69^*^	36	34	4.4 (3.1-5.1)		1.00		36	29	8.7 (6.5-16.8)		1.00	
≥69	39	36	3.3 (2.1-4.7)	0.707	0.91 (0.56-1.48)	0.707	39	24	10.7 (7.6-15.3)	0.313	0.75 (0.43-1.31)	0.315
**Debulking surgery**												
No	9	8	4.7 (0.9-21.9)		1.00		9	6	19.4 (1.5-nr)		1.00	
Yes	66	62	3.4 (2.9-4.9)	0.992	1.00 (0.47-2.10)	0.992	66	47	9.5 (8.0-14.1)	0.857	0.92 (0.39-2.18)	0.857
**FIGO stage**												
I-II	9	9	8.7 (1.6-44.1)		1.00		9	7	24.1 (2.6-nr)		1.00	
III	43	41	4.3 (3.1-4.9)		0.44 (0.19-1.02)		43	29	10.6 (7.6-16.8)		0.76 (0.33-1.77)	0.527
IV	23	20	2.5 (1.8-6.4)	0.090	1.15 (0.67-1.97)	0.103	23	17	8.4 (3.6-12.9)	0.174	1.61 (0.87-2.97)	0.182
**ECOG PS**												
0	34	32	4.3 (3.1-5.1)		1.00		34	25	12.0 (8.0-23.2)		1.00	
1	32	30	3.3 (2.1-4.7)	0	1.19 (0.72-1.97)		32	22	8.4 (3.8-16.8)		1.59 (0.88-2.88)	0.121
2-3	8	7	4.8 (0.9-nr)	0.796	1.13 (0.49-2.58)	0.777	8	6	8.4 (1.1-nr)	0.133	2.20 (0.88-5.55)	0.093
**Pre-treatment Ca125**												
<35^*^	8	7	5.6 (1.8-nr)		1.00		32	27	7.2 (4.0-10.6)		1.00	
≥35	63	59	3.4 (2.5-4.5)	0.081	2.09 (0.89-4.89)	0.089	32	19	16.8 (8.8-24.1)		0.53 (0.29-0.97)	
**Pre-treatment Hb**							11	7	9.0 (3.5-nr)	0.096	0.62 (0.27-1.44)	0.103
>12.5^#^	18	17	4.7 (2.4-7.0)		1.00							
≤12.5	52	48	3.4 (2.6-4.6)	0.176	1.49 (0.83-2.69)	0.181	8	5	10.6 (6.5-nr)		1.00	
**Pre-treatment NLR**							63	44	8.8 (7.2-14.1)	0.317	1.68 (0.60-4.70)	0.323
<3	34	33	3.0 (2.1-4.3)		1.00							
≥3	33	29	4.4 (2.5-6.4)	0.271	0.75 (0.45-1.25)	0.274	18	15	8.7 (6.4-17.9)			
**Pre-treatment PLR**							52	34	11.5 (7.6-16.5)	0.758	0.91 (0.49-1.68)	0.758
<210	34	32	3.9 (2.6-6.4)		1.00							
≥210	34	31	3.3 (1.9-4.5)	0.542	1.17 (0.71-1.93)	0.544	34	22	10.6 (7.2-15.3)		1.00	
**Starting dose of melphalan**							33	25	8.4 (3.8-16.8)	0.650	1.15 (0.64-2.06)	0.650
95-100%	32	30	3.3 (2.1-5.0)		1.00							
75-94%	32	30	4.6 (2.9-6.8)		0.71 (0.43-1.20)		34	22	10.7 (8.3-17.9)		1.00	
<75%	11	10	2.9 (0.9-8.0)	0.432	0.90 (0.43-1.86)	0438	34	26	8.0 (3.7-14.1)	0.114	1.59 (0.89-2.84)	0.117
**BRCA status**												
Wild-type	25	24	2.6 (1.9-4.4)		1.00		25	16	8.0 (4.0-12.0)		1.00	
Mutated	11	8	6.2 (3.7-nr)	0.001	0.25 (0.10-0.61)	0.002	11	7	25.9 (3.7-nr)	0.086	0.38 (0.12-1.19)	0.097

^*^Median value; **^#^**upper normal value.

CI, confidence interval; ECOG, Eastern Cooperative Oncology Group; FIGO, International Federation of Gynecology and Obstetrics; Hb, hemoglobin; HR, hazard ratio; mo, months; N, number; NLR, neutrophil-to-lymphocyte ratio; nr=not reached; PLR, platelet-to-lymphocyte ratio; PFS, progression-free survival; PS, performance status; pts, patient.

We studied the association of BRCA1/2 status with melphalan treatment. In EOC patients with available molecular data, when comparing baseline characteristics of BRCA1/2 mutant to BRCA1/2 wild type patients, no differences were observed (**Supplementary Table 1**).

Based on BRCA mutational status, we reported in 11 BRCA1/2 mutated patients an overall response rate (ORR) (CR+PR) of 18.2%, SD and PD in 36.4% and 45.4% of cases, respectively; whereas 25 BRCA1/2 wild type patients had ORR of 4%, SD and PD in 36% and 60% of cases, respectively.

**Figure 2:**
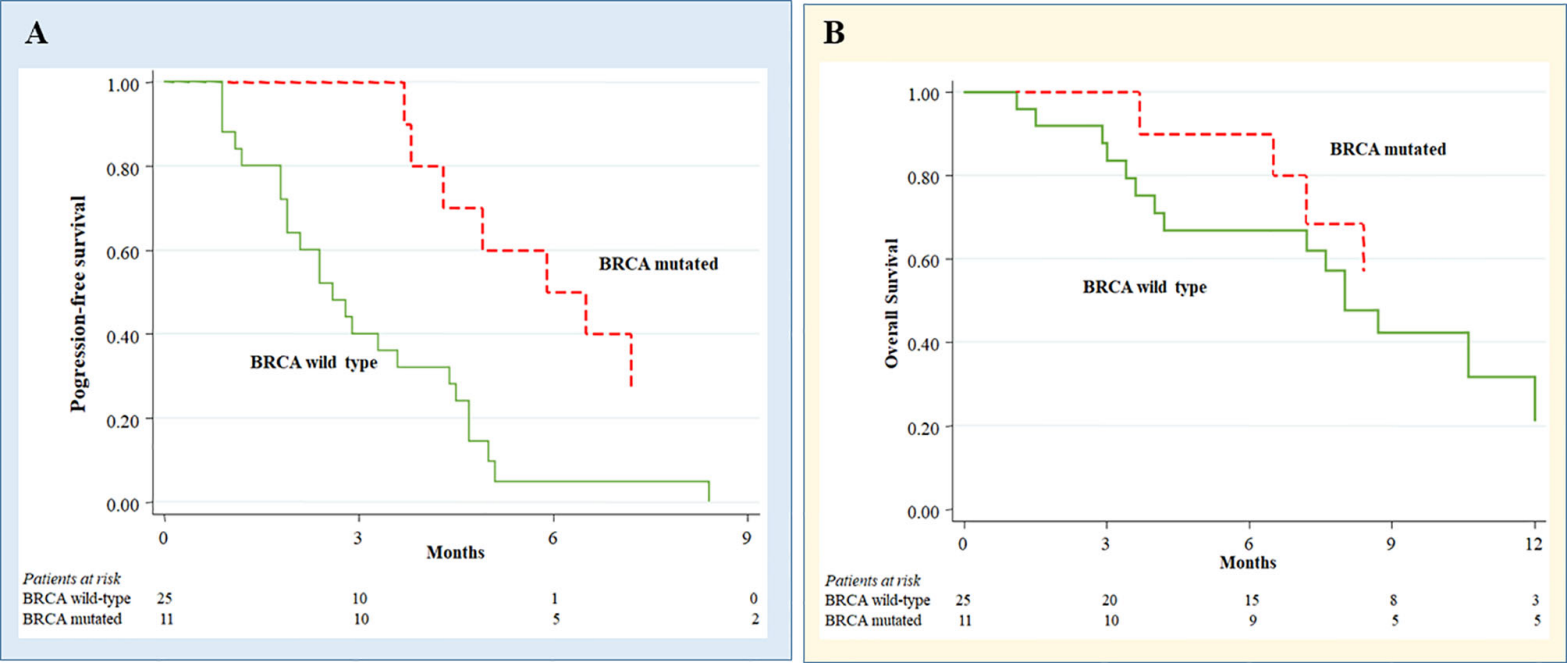
Melphalan treatment outcomes in ovarian cancer patients according to BRCA status. Progression-free survival **(A)** and Overall survival **(B)** in melphalan-treated ovarian cancer patients according to BRCA status.

In BRCA1/2 mutant EOC receiving melphalan, we observed a significant longer PFS compared to BRCA1/2 wild type patients (median, 6.2 *versus* 2.6 months; hazard ratio (HR) 0.25, 95% confidence interval (CI) 0.10-0.61; *p* = 0.002) (**Figure 2A**). Moreover, a trend was seen for BRCA1/2 mutants to have a better OS (median, 25.9 *versus* 8.0 months; HR 0.38; 95% CI 0.12-1.19; *p* = 0.097) (**Figure 2B**).

### Safety and Tolerability

Thirty-two patients (42.7%) were treated with initial standard melphalan regimen. In 21 patients (28%), the dose of melphalan was reduced by at least 75%, and in more than 80% of cases, reduction was required because of hematologic toxicity. However, only 4 (5.3%) patients receiving melphalan discontinued treatment because of unacceptable toxicity (two grade 3 thrombocytopenia, one grade 2 anemia, and one grade 4 neutropenia).

The hematological and non-hematological adverse events occurred in our series are reported in **Table 3**. As expected, myelotoxicity was the prevalent toxicity with 17.3% of patient experiencing grade 3-4 thrombocytopenia, 13.4% and 6.7% of patients reporting grade 3-4 neutropenia and anemia, respectively. Nausea and vomiting and fatigue were the most frequent non-hematologic events (grade 3: 2.7% and 4% of patients, respectively). Neither cardiac and renal toxicities nor treatment-related deaths were reported.

**Table 3:** Toxicity in melphalan-treated patients.

Toxicity	Grade 1 N (%)	Grade 2 N (%)	Grade 3 N (%)	Grade 4 N (%)
Anemia	3 (4)	8 (10.7)	5 (6.7)	0 (0)
Neutropenia	9 (12.0)	3 (4)	8 (10.7)	2 (2.7)
Thrombocytopenia	1 (1.3)	7 (9.3)	12 (16.0)	1 (1.3)
Nausea/vomiting	2 (2.7)	2 (2.7)	2 (2.7)	0 (0)
Fatigue	5 (6.7)	5 (6.7)	1 (1.3)	0 (0)
Diarrhea	2 (2.7)	2 (2.7)	0 (0)	0 (0)
Mucositis	0 (0)	1 (1.3)	2 (2.7)	0 (0)
Liver	0 (0)	2 (2.7)	0 (0)	0 (0)

There was no difference of hematological toxicity related to melphalan treatment between BRCA mutants *versus* wild-type patients.

## Discussion

Our study represents the largest cohort of EOC patients treated with melphalan reported to date. Currently, the management of platinum-resistant EOC represents one of the most important unmet medical issues. Despite great research endeavors over the last decades, standard treatments have often inadequate clinical benefit. Here, we report a considerable clinical activity of melphalan chemotherapy in this difficult-to-treat patients group. For exploratory purposes, we also investigated a subset of BRCA1/2 mutated patients showing improved outcomes and enhanced sensitivity to melphalan chemotherapy compared to BRCA1/2 wild type women.

An overall clinical benefit of 58.7% in a population who has received a median of four previous chemotherapy lines appears as a notable result. In heavily-pre-treated platinum-resistant EOC patients with a median of four prior therapeutic lines, the overall clinical benefit observed in this work was slightly higher than that observed in our previous retrospective experience of women receiving FOLFOX-4 and topotecan as salvage chemotherapy lines (30.8% and 48.3%, respectively) (10). A multicenter retrospective study showed that weekly paclitaxel monochemotherapy had similar clinical benefit rate of 36%, with a median PFS of 21 weeks (18), which is consistent with the PFS of 4.7–5.3 months in the SaPPrOC trial (19). Both these studies suggested a therapeutic role of weekly paclitaxel in patients with EOC regardless of BRCA1/2 status. Similar evidence was reported in the prospective MITO-15 phase II trial (20) in which trabectedin showed a very similar clinical benefit (54.2%) to melphalan, and no differences in treatment outcomes were observed according to BRCA1/2 status (20).

According to these findings, the outcomes of melphalan treatment were quite similar to those reported with other drugs for heavily pre-treated platinum-resistant EOC patients. However, there is increasing evidence to consider BRCA mutation status when selecting not only PARPi agents but also chemotherapy regimens, such as melphalan treatment. In support of this conjecture, melphalan was shown to be selectively toxic to BRCA2-deficient breast cancer cell lines and to produce a longer relapse-free survival in mice than platinum or olaparib (21).

Overall, we recognize some limitations of our study such as the relatively modest sample size, influencing the statistical significance especially for OS, the clinical and histological heterogeneity of the patients’ cohort and its retrospective, non-randomized design. In addition, several studies showed that BRCA mutant patients have, in general, better prognosis, likely due to the high response rate to platinum-based chemotherapy. This aspect could be a confounding factor in the interpretation of our survival data in melphalan-treated patients according to BRCA status; however, the exact effect of BRCA1/2 mutations on EOC prognosis is still controversial (22–24). Lastly, we have considered only BRCA1/2 mutations and not other alterations in DNA defect repair genes and no patient did prior therapy with PARPi. Nevertheless, our preliminary results suggest that BRCA status is associated with sensitivity to melphalan therapy. Thus, since BRCA-related EOC represents a distinct entity within the ovarian cancer spectrum, developing a subtype-specific treatment tailored to the unique cancer biology of ‘BRCA-pathway’ ovarian tumours (arising from germ-line or somatic BRCA mutations) may lead to an improved disease management. Some preclinical studies demonstrated a decreased likelihood of response to subsequent chemotherapy following olaparib treatment due to the development of cross-resistance between PARPi and platinum-based regimens through the acquisition of secondary mutations restoring BRCA1/2 protein expression (25, 26). In this context, the availability of other active DNA alkylating agents could constitute an additional therapeutic option for resistant or recurrent EOC BRCA1/2 mutant patients.

## Conclusion

In heavily pre-treated EOC patients, melphalan chemotherapy is an effective and well-tolerated treatment. Discovering the underlying molecular mechanism of chemo-responsiveness could lead to subtype-specific treatment selection. This study supports the notion that the knowledge on BRCA status may improve clinical decision-making in choosing between different therapies for platinum-resistant EOC. Prospective trials including overall BRCA/Homologous recombination deficiency assessment are warranted.

## Data Availability Statement

The raw data supporting the conclusions of this article will be made available by the authors, without undue reservation.

## Ethics Statement

The studies involving human participants were reviewed and approved by COMITATO ETICO della Romagna. The patients/participants provided their written informed consent to participate in this study.

## Author Contributions

VC was involved in the conception of the study, acquisition and analysis of the data, and wrote the first draft of the manuscript. AF, NB, LR, GG, CL, GS, SB, CG, AV, AA, SLB, and CM were involved in the acquisition of the data. VC and UDG were involved in the conception and design of the study. VC, ES, and UDG contributed to data analysis and interpretation of data. VC, ES, AA, NB, and UDG critically revised the manuscript for important intellectual content. VC and ES participated in analyzing the results and drafting the manuscript. All authors contributed to the article and approved the submitted version.

## Conflict of Interest

VC has served as consultant/advisory board member for Janssen, Astellas, Merck, AstraZeneca, Bayer; has received speaker honoraria or travel support from Astellas, Janssen, Ipsen, Bayer and Sanofi. UDG reports research support from AstraZeneca, Roche, and Sanofi; and consultancy fees from Astellas, Bayer, Bristol Myers Squibb, Ipsen, Janssen, Merck, Pfizer, and Sanofi.

The remaining authors declare that the research was conducted in the absence of any commercial or financial relationships that could be construed as a potential conflict of interest.
