# Supplementary material for: Melphalan as a Promising Treatment for BRCA-Related Ovarian Carcinoma
Source: Front Oncol. 2021 Jul 21;11:716467. doi: 10.3389/fonc.2021.716467 (PMC8336462; doi:10.3389/fonc.2021.716467)
Supplement: Supplementary file 1 [file Table_1.docx]

**Supplementary Table 1. Patient characteristics according to BRCA status**

|  | **BRCA** | | |  | |
| --- | --- | --- | --- | --- | --- |
|  | | **Wild type**  N (%) | **Mutated**  N (%) | | **p** |
| **Median age**, years (range) | | 70 (45-83) | 62 (33-71) | | 0.063 |
| **FIGO stage at diagnosis** | |  |  | |  |
| I-II | | 3 (12.0) | 0 | |  |
| III | | 14 (56.0) | 6 (54.5) | |  |
| **Primary debulking surgery** | |  |  | |  |
| No | | 5 (20.0) | 2 (18.2) | |  |
| Yes | | 20 (80.0) | 9 (81.8) | | 0.900 |
| **ECOG PS** | |  |  | |  |
| 0-1 | | 22 (88.0) | 10 (90.9) | |  |
| ≥2 | | 3 (12.0) | 1 (9.1) | | 0.475 |
| **Median baseline CA-125**, ng/mL (range) | | 194.9  (11.1-3319) | 249.65  (19.5-10535) | | 0.719 |
| **Pre-treatment aemoglobin**, g/dL | |  |  | |  |
| >12.5^#^ | | 5 (22.7) | 3 (27.3) | |  |
| ≤12.5 | | 17 (77.3) | 8 (72.7) | | 0.773 |
| **Pre-treatment NLR** | |  |  | |  |
| <3 | | 14 (63.6) | 4 (40.0) | |  |
| ≥3 | | 8 (36.4) | 6 (60.0) | | 0.219 |
| **Pre-treatment PLR** | |  |  | |  |
| <210 | | 10 (45.5) | 4 (40.0) | |  |
| ≥210 | | 12 (54.5) | 6 (60.0) | | 0.777 |
| **Median number of cycles of Alkeran** (range) | | 3 (1-6) | 3 (1.15) | | 0.930 |
| **Starting dose of melphalan** | |  |  | |  |
| 95-100% | | 9 (36.0) | 5 (45.5) | |  |
| 75-94% | | 12 (48.0) | 5 (45.5) | |  |
| <75% | | 4 (16.0) | 1 (9.0) | | 0.513 |
| **Dose reduction of Melphalan during treatment** | | | | | |
| No | | 22 (91.7) | 8 (80.0) | |  |
| Yes | | 2 (8.3) | 2 (20.0) | | 0.343 |

^#^ Upper normal value

*Abbreviations.* ECOG, Eastern Cooperative Oncology Group; FIGO International Federation of Gynecology and Obstetrics; N, number; NLR, neutrophil-to-lymphocyte ratio; PLR platelet-to-lymphocyte ratio; PS, performance status.
